# Foot characteristics during walking in 6–14- year-old children

**DOI:** 10.1038/s41598-020-66498-5

**Published:** 2020-06-11

**Authors:** Mario Kasović, Lovro Štefan, Martin Zvonár

**Affiliations:** 1 Department of General and Applied Kinesiology, Faculty of Kinesiology, University of Zagreb, Zagreb, Croatia; 2 Department of Sport Motorics and Methodology in Kinathropology, Faculty of Sports Studies, Masaryk University, Brno, Czech Republic; 3 Faculty of Science, Masaryk University, 62500 Brno, Czech Republic

**Keywords:** Health occupations, Risk factors

## Abstract

The main purpose of the study was to establish foot characteristics during walking in children. In this cross-sectional study, we recruited 1 284 primary-school students aged 6–14 years (714 boys and 570 girls) randomly selected from five schools in the city of Brno, Czech Republic. Children walked across a pressure platform (EMED-xl; Novel_GmbH_, Munich, Germany) to collect the data for both left and right foot during three trials. After the procedure, the software generated several foot characteristic variables: (1) force-time integral, (2) pressure-time integral, (3) contact area, (4) contact time, (5) peak pressure and (6) average pressure for the total foot. Curves for the 5^th^, 10^th^, 25^th^, 50^th^, 75^th^, 90^th^ and 95^th^ percentiles were calculated using the Lambda, Mu and Sigma (LMS) Chartmaker software. Our results showed that boys had longer force-time integral, higher contact area and contact time values, and higher peak plantar pressure, while no significant differences in pressure-time integral and average plantar pressure between sexes were observed. Older boys and girls had higher values in all measured variables. Our results provide for the first-time sex- and age-specific foot characteristics during walking in 6–14-year-old children.

## Introduction

Foot characteristics in children have become well-studied in the past 15 years. Some of them, like plantar pressures, represent the force applied to the ground and its distribution over the foot plantar surface area^1^. According to previous evidence, peak vertical ground reaction forces may generate up to 120% of body weight^2^, where several tenth or hundred tones can be absorbed by each foot^3^. Since walking is one of the main biological needs of individuals, constant high loadings lead to pain and discomfort in the region of lower extremities^4,5^. Such conditions have been proven to effect on health-related factors, reducing the level of physical activity^5^ or gaining weight^6^.

Children are at vulnerable risk for developing acute and chronic foot diseases^7^_,_ since their feet still have immature structure and are under developmental phase^8^. From a biological point of view, children experience normal growth and have flexible flat feet^9^. Despite that, studies have shown that parents are frequently worried about their children’s feet and seek additional medical attention^9–11^. The most common external factor influencing normal foot structure is ill-fitting shoes, which often deviate from normal function and increase plantar pressure distribution^12^. Moreover, previous evidence has suggested that foot pain and discomfort have been associated with higher levels of plantar pressures in adults^13,14^, leading to the conclusion that force and pressure beneath the foot are important determinants of the foot. Thus, including plantar pressure variables within health surveillance systems should be of important interest for health-related professions to screen appropriate loading and temporal properties of the foot.

After an extensive literature review, we found no studies that have established foot characteristics in children. Therefore, the main purpose of the study was to establish sex- and age-specific foot characteristics data for several plantar pressure variables in 6–14-year-old children.

## Materials and Methods

### Study participants

In this cross-sectional study, participants were 1 284 primary-school children (6–14 years (age_mean ± SD_ = 9.6 ± 2.3; 44.4% girls) randomly chosen from five public schools in the city of Brno, Czech Republic. The inclusion criteria were: (1) age 6–14 years, (2) without history of any diseases, (3) currently without any diseases and (4) attending school and class where the study was conducted. At the first stage, we contacted principals from each school to give permission for conducting the study. At the second stage, we introduced children and their parents with measurement protocol, potential contribution of the research, and possible discomforts during the execution of the research. Those children whose’ parents had given written informed consent entered the study. All procedures were in accordance with the Declaration of Helsinki and approved by the Committee of the Faculty of Sports Studies (Ethics code number: 2/2018).

### Dynamic foot characteristics variables

Dynamic plantar distributions generated under left and right foot were quantified as the children walked over a calibrated EMED – XL pressure platform (frequency of 100 Hz, resolution of 4 sensors/cm^2^, 1440 ×440 mm sensor area and pressure range between 10–1270 kPa; Novel_gmbh_, Munich, Germany). Children were asked to normally walk over the platform with previous familiarization, as done in previous studies^8^. In brief, an adult assisted each participant by holding their hand during walking over the platform, after which the adult walked beside the participants without holding the hand to ensure normal arm swing. Software generated the data for three successful trials for both feet as follows: (1) force - time integral (N*s), (2) pressure - time integral (kPa*s), (3) contact area (cm^2^), (4) contact time (ms), (5) peak pressure (kPa) and (6) mean pressure (kPa). Of note, force-time integral and pressure-time integral are variables which describe the cumulative effect of force and pressure over time in a certain area of the foot, additionally providing a value for the total load exposure of a foot sole area during one step^15^. Of note, previous studies have shown that cumulative effect of force and pressure can lead to tissue damage and increase the risk of skin trauma^15^. Contact area is defined as the area covered by foot during one step and contact time is defined as the time interval between initial ground contact and toe off. Normal foot tends to have larger contact area covered and shorter contact time, which in addition leads to less rigid and more stable foot to absorb impact^16^. Peak and average plantar pressures represent the maximal and average load in an area under the foot during one step^17^. Sex and age were collected prior the foot measurement protocol. The presence of foot pain was assessed by a single-item question:’Did you experience foot pain in one or both feet in the last 30 days’ with ‘Yes’ and ‘No’ answers.

### Data analysis

Basic descriptive statistics are presented as mean (x) and standard deviation (SD). Sex and age differences were calculated by using analysis of variance (ANOVA) with *post hoc* comparison test between the groups. To calculate correlations between age and all the study variables, we used Pearson coefficient of correlation (r). For each variable, we determined sex- and age- specific percentile values (5^th^, 10^th^, 25^th^, 50^th^, 75^th^, 90^th^ and 95^th^) and used Cole’s Lambda, Mu and Sigma (LMS) method, in which the optimal power to obtain normality is summarized by a smooth (L) curve and trends in the mean (M) and coefficient of variation (S) are similarly smoothed^18^. Next, all three curves (L, M and S) are summarized based on the power of age-specific Box–Cox power transformations for normalizing the data^18^. All analyses were performed in Statistical Packages for Social Sciences (SPSS Inc., Chicago, Illinois, USA) and in LMS Chartmaker Pro version (The Institute of Child Health, London, UK). A p value of <0.05 (two sided) was considered statistically significant.

## Results

Basic descriptive statistics of the study participants are presented in Table 1. Boys were slightly older, compared to girls (p = 0.039). Boys also had higher force-time integral values, followed by higher contact time, contact area and peak pressure values for both feet. No significant differences between sexes in terms of pressure-time integral and average pressure for left foot were observed (p > 0.05). Of note, we found statistically significant differences between left and right foot in terms of force-time integral (t = −5.68, df=1283, p < 0.001), contact area (t = −5.404, df=1283, p < 0.001), contact time (t = −3.303, df=1283, p < 0.001) and average plantar pressure (t = −2.401, df=1283, p = 0.017), so we presented upcoming results specifically for left and right foot.

**Table 1:** Basic descriptive statistics of the study participants, Czech Republic.

Study variables	Total (N = 1 284)	Boys (N = 714)	Girls (N = 570)	p-value*
	x (SD)	x (SD)	x (SD)	
Age (years)	9.6 (2.3)	9.8 (2.3)	9.5 (2.3)	0.039
Force-time integral (N*s; left foot)	194.7 (82.9)	203.7 (90.1)	183.5 (71.5)	<0.001
Force-time integral (N*s; right foot)	196.4 (83.9)	205.1 (91.2)	185.6 (72.4)	<0.001
Pressure-time integral (kPa*s; left foot)	139.3 (45.0)	140.1 (45.9)	138.4 (43.8)	0.513
Pressure-time integral (kPa*s; right foot)	139.2 (50.8)	138.2 (45.3)	140.5 (56.9)	0.416
Contact area (cm^2^; left foot)	110.5 (21.9)	113.9 (23.3)	106.1 (19.1)	<0.001
Contact area (cm^2^; right foot)	111.5 (21.8)	114.9 (23.4)	107.1 (18.7)	<0.001
Contact time (ms; left foot)	631.9 (82.9)	643.3 (88.3)	617.7 (73.2)	<0.001
Contact time (ms; right foot)	634.6 (84.0)	644.9 (89.9)	621.8 (74.1)	<0.001
Peak pressure (kPa; left foot)	423.0 (130.9)	414.9 (129.1)	433.3 (132.7)	0.012
Peak pressure (kPa; right foot)	419.1 (131.3)	404.3 (127.4)	437.7 (133.9)	<0.001
Average pressure (kPa; left foot)	86.2 (13.7)	85.7 (13.4)	86.9 (14.0)	0.124
Average pressure (kPa; right foot)	86.8 (15.3)	85.9 (13.5)	88.0 (17.2)	0.016

*differences were calculated using Student t-test for independent (sex) samples.

p < 0.05.

Table 2 shows sex- and age-specific foot characteristics for left and right foot in variables force- and pressure-time integrals. The median value for force-time integral was between 108 N*s at age 6 to 348 N*s at age 14 in boys. In girls, similar patterns to boys in force-time integral till the age of 10 were observed, after which the differences to the age of 14 became larger. The median value for pressure-time integral ranged between 93 and 186 kPa*s in boys and between 95 and 179 kPa*s in girls, respectively. Similar patterns between sex and sex*age interaction were observed (p > 0.05). Chronological age was significantly correlated with force-time integral and pressure-time integral (r = 0.50–0.80, p < 0.001).

**Table 2:** Foot characteristics for left and right foot (L/R within the table) in force– and pressure– time integrals.

Measure	Sex	Age	N	P5	P10	P25	P50	P75	P90	P95
Force-time integral	Boys	6	38	77/76	80/83	89/94	108/111	126/130	143/151	155/161
		7	92	90/91	95/95	107/110	125/127	141/143	161/155	167/166
		8	130	106/107	114/113	126/127	148/149	168/169	189/194	228/216
		9	116	118/117	131/129	151/149	168/167	195/196	229/224	256/269
		10	70	126/123	134/135	152/154	173/175	207/206	234/237	261/257
		11	64	144/147	156/161	186/191	222/227	264/254	300/302	337/343
		12	94	162/161	175/177	218/219	252/258	319/317	364/371	383/391
		13	48	206/204	235/240	273/278	309/305	362/361	416/420	470/465
		14	62	230/238	258/259	305/295	342/348	393/393	481/480	595/560
	Girls	6	58	78/75	82/81	93/95	110/112	133/135	159/171	200/200
		7	80	95/98	101/100	110/110	128/128	143/143	172/171	185/185
		8	82	100/99	104/108	118/119	134/134	160/162	185/188	210/229
		9	68	112/113	122/123	142/148	169/172	199/200	244/243	256/259
		10	98	115/116	125/127	143/147	174/174	202/207	236/246	276/291
		11	60	148/155	158/156	177/177	195/198	229/228	267/267	296/307
		12	48	174/172	184/187	215/212	250/253	277/283	300/316	320/333
		13	38	154/152	174/176	211/211	275/269	324/337	346/355	537/535
		14	38	232/224	239/224	261/277	294/299	336/343	417/415	433/428
Pressure-time integral	Boys	6	38	74/62	76/66	81/85	95/93	111/108	132/141	173/187
		7	92	74/72	80/80	95/91	103/99	120/115	136/158	158/163
		8	130	81/77	84/84	94/95	116/112	136/132	167/153	178/166
		9	116	89/86	94/93	104/105	122/119	138/138	158/157	167/175
		10	70	88/92	96/101	119/113	138/136	158/150	182/177	210/220
		11	64	86/91	112/106	128/125	153/150	183/172	211/220	241/236
		12	94	113/110	119/127	141/139	162/156	189/197	207/225	230/231
		13	48	129/127	135/130	152/146	174/162	198/196	223/231	228/272
		14	62	125/131	140/143	159/161	186/186	236/210	273/262	357/286
	Girls	6	58	74/76	80/81	87/86	96/95	110/107	127/124	144/147
		7	80	78/80	83/83	91/95	108/107	128/123	147/150	165/165
		8	82	77/79	83/84	94/101	112/116	131/130	148/154	162/166
		9	68	92/92	95/97	112/113	132/131	156/150	181/170	191/189
		10	98	89/90	97/101	118/115	134/135	167/159	201/188	214/211
		11	60	100/105	111/119	131/135	156/154	192/192	237/224	272/247
		12	48	118/120	122/129	141/136	151/151	175/174	222/241	250/273
		13	38	92/105	114/125	147/157	180/177	209/213	239/252	258/279
		14	38	108/129	132/134	152/154	179/177	202/214	245/287	282/327

Table 3 shows sex- and age-specific foot characteristics for left and right foot in variables contact area and contact time. The median value for contact area was between 92 and 147 cm^2^ in boys. In girls, the median value ranged between 86 and 127 cm^2^. As expected, boys had significantly higher contact area values, compared to girls. The median value for contact time ranged between 570 and 720 ms in boys and between 580 and 692 ms in girls. Chronological age was significantly correlated with contact area and contact time (r = 0.37–0.74, p < 0.001).

**Table 3:** Foot characteristics for left and right foot (L/R within the table) in contact area and contact time.

Measure	Sex	Age	N	P5	P10	P25	P50	P75	P90	P95
Contact area	Boys	6	38	74/71	76/75	86/86	92/94	98/99	102/103	103/105
		7	92	76/74	77/80	87/89	94/95	100/101	108/109	115/115
		8	130	85/85	87/88	93/94	101/101	110/110	119/116	124/125
		9	116	84/79	88/88	98/98	110/109	118/119	126/128	129/133
		10	70	78/81	82/86	99/101	107/110	118/117	131/130	140/143
		11	64	92/86	97/99	107/110	116/116	134/132	141/145	147/150
		12	94	97/105	106/110	119/120	129/129	140/140	156/155	175/171
		13	48	114/117	127/129	139/139	144/147	158/155	169/172	172/179
		14	62	120/122	122/125	129/131	145/146	162/160	177/181	182/186
	Girls	6	58	67/69	68/72	74/77	85/86	94/96	107/106	110/113
		7	80	71/72	77/77	86/85	95/94	100/101	109/112	120/119
		8	82	74/77	81/84	91/91	98/98	106/107	115/114	123/122
		9	68	80/83	85/87	94/94	106/109	118/118	124/123	132/129
		10	98	86/87	91/92	98/100	106/106	115/118	126/127	128/131
		11	60	96/94	98/101	104/106	111/112	117/119	132/126	134/131
		12	48	98/99	101/100	109/105	121/122	134/133	142/145	147/147
		13	38	94/89	99/105	108/111	127/126	145/148	155/161	172/163
		14	38	100/105	104/108	114/116	132/134	139/142	150/152	154/156
Contact time	Boys	6	38	450/454	490/509	525/543	570/585	612/641	734/721	835/854
		7	92	489/486	503/505	557/560	600/597	637/637	691/685	761/736
		8	130	504/504	520/530	559/553	600/600	653/663	720/709	771/767
		9	116	509/503	532/523	570/573	620/610	660/663	703/711	740/758
		10	70	524/517	540/543	563/569	620/617	663/674	706/710	729/736
		11	64	538/534	578/568	611/617	640/647	676/673	738/748	786/783
		12	94	565/565	597/602	642/650	693/690	743/748	790/803	836/823
		13	48	621/610	642/642	678/684	720/720	756/750	793/810	840/850
		14	62	643/638	647/644	672/679	710/713	758/763	836/868	904/900
	Girls	6	58	473/467	492/490	517/519	580/583	625/631	673/687	708/708
		7	80	494/490	513/515	541/554	587/598	642/644	713/705	763/757
		8	82	500/500	519/510	540/550	578/588	628/640	687/717	716/742
		9	68	506/516	520/543	563/557	587/592	647/656	721/704	749/744
		10	98	510/520	523/533	556/569	610/618	651/657	693/684	707/707
		11	60	531/537	564/570	588/591	620/628	681/672	719/722	733/740
		12	48	598/603	606/613	641/638	665/668	715/696	764/759	767/786
		13	38	516/510	563/568	601/607	655/640	688/683	749/762	771/791
		14	38	606/597	610/603	642/647	680/692	730/733	771/777	794/801

Table 4 shows sex- and age-specific foot characteristics for left and right foot in variables peak pressure and average pressure. The median value for peak pressure was between 304 and 465 kPa in boys and between 350 and 516 kPa in girls. Interestingly, girls generated higher peak pressure between ages 6 and 9, had similar values at the age of 10 and continued to have higher values till the age of 14. The median value for average pressure ranged between 70 and 102 kPa in boys and between 75 and 104 kPa in girls, respectively. Although we found no significant differences between sexes in average pressure for left foot (p = 0.124), girls generated significantly higher average pressure for right foot, compared to boys (p = 0.016). As for the previous variables, chronological age was significantly correlated with peak pressure and average pressure (r = 0.32–0.63, p < 0.001). Of note, we additionally calculated foot asymmetries between left and right foot and sex*age interactions of the studied variables and found no significant differences (asymmetry index 2%-8%, p > 0.05 and sex*age interaction p > 0.05).

**Table 4:** Foot characteristics for left and right foot (L/R within the table) in peak pressure and average pressure.

Measure	Sex	Age	N	P5	P10	P25	P50	P75	P90	P95
Peak pressure	Boys	6	38	236/206	245/223	281/263	304/318	354/375	439/426	494/518
		7	92	210/203	232/234	290/265	349/330	403/410	456/554	549/616
		8	130	228/206	255/237	295/288	363/349	454/437	575/530	632/585
		9	116	243/250	265/271	305/308	380/368	443/442	511/524	537/560
		10	70	269/257	297/297	357/355	430/407	500/508	689/665	755/733
		11	64	276/276	309/295	379/357	423/420	527/486	591/590	697/702
		12	94	290/271	312/288	355/353	432/430	535/549	613/597	690/693
		13	48	281/287	303/310	381/344	445/406	515/482	600/642	807/726
		14	62	307/330	340/342	377/391	450/465	593/525	730/676	894/791
	Girls	6	58	221/217	260/229	283/295	350/352	426/392	453/444	507/480
		7	80	253/233	259/262	296/303	363/372	455/481	526/580	636/692
		8	82	220/245	229/264	300/303	382/372	447/468	550/625	642/726
		9	68	276/264	310/311	360/378	427/441	480/472	566/544	646/568
		10	98	291/288	313/315	362/363	425/421	519/503	655/621	694/724
		11	60	320/296	353/352	391/405	473/497	600/583	713/666	755/690
		12	48	289/292	315/313	353/383	432/444	543/507	603/705	648/749
		13	38	287/314	332/350	385/400	516/513	705/589	800/799	866/891
		14	38	275/290	331/338	382/365	459/470	550/599	609/730	645/761
Average pressure	Boys	6	38	59/60	60/61	66/67	70/71	74/75	79/78	81/84
		7	92	60/60	64/63	69/68	75/75	81(82	86/89	88/94
		8	130	65/64	67/67	73/73	78/78	87(86	94/93	99/99
		9	116	69/68	70/70	77/76	83/83	92/92	101/99	105/108
		10	70	71/72	75/75	79/81	87/87	94/97	103/102	106/106
		11	64	73/76	76/79	84/83	89/91	100/100	106/107	112/111
		12	94	78/76	79/79	85/84	90/91	98/99	108/109	113/114
		13	48	77/71	80/76	87/84	93/95	101/102	109/111	119/113
		14	62	85/84	89/87	93/94	102/101	110/109	116/120	123/127
	Girls	6	58	61/62	63/65	71/71	76/75	81/82	88/87	91/87
		7	80	64/62	66/67	72/72	77/78	83/84	91/92	97/102
		8	82	64/64	65/69	71/73	79/81	87/90	93/99	99/104
		9	68	68/74	72/75	79/79	88/88	93/96	99/99	102/103
		10	98	70/72	73/76	80/80	88/86	95/94	102/104	109/109
		11	60	76/73	77/75	84/85	93/92	101/101	113/115	123/122
		12	48	74/74	75/80	87/86	92/93	102/102	107/108	115/120
		13	38	81/80	83/83	93/93	103/104	111/108	119/118	138/148
		14	38	90/90	92/92	98/96	104/103	112/110	116/116	121/117

**Figure 1:**
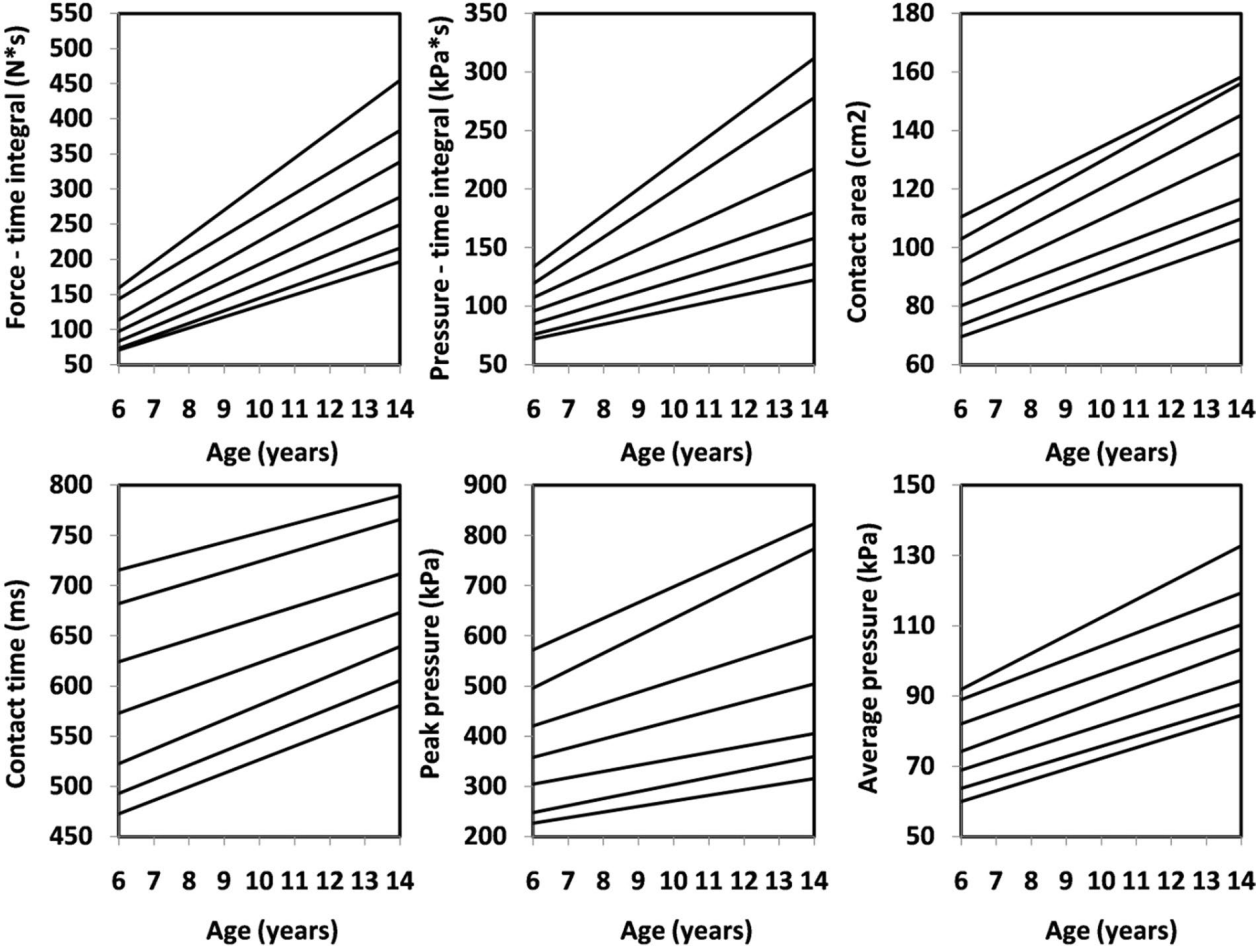
Percentile values for foot function (left) in boys.

**Figure 2:**
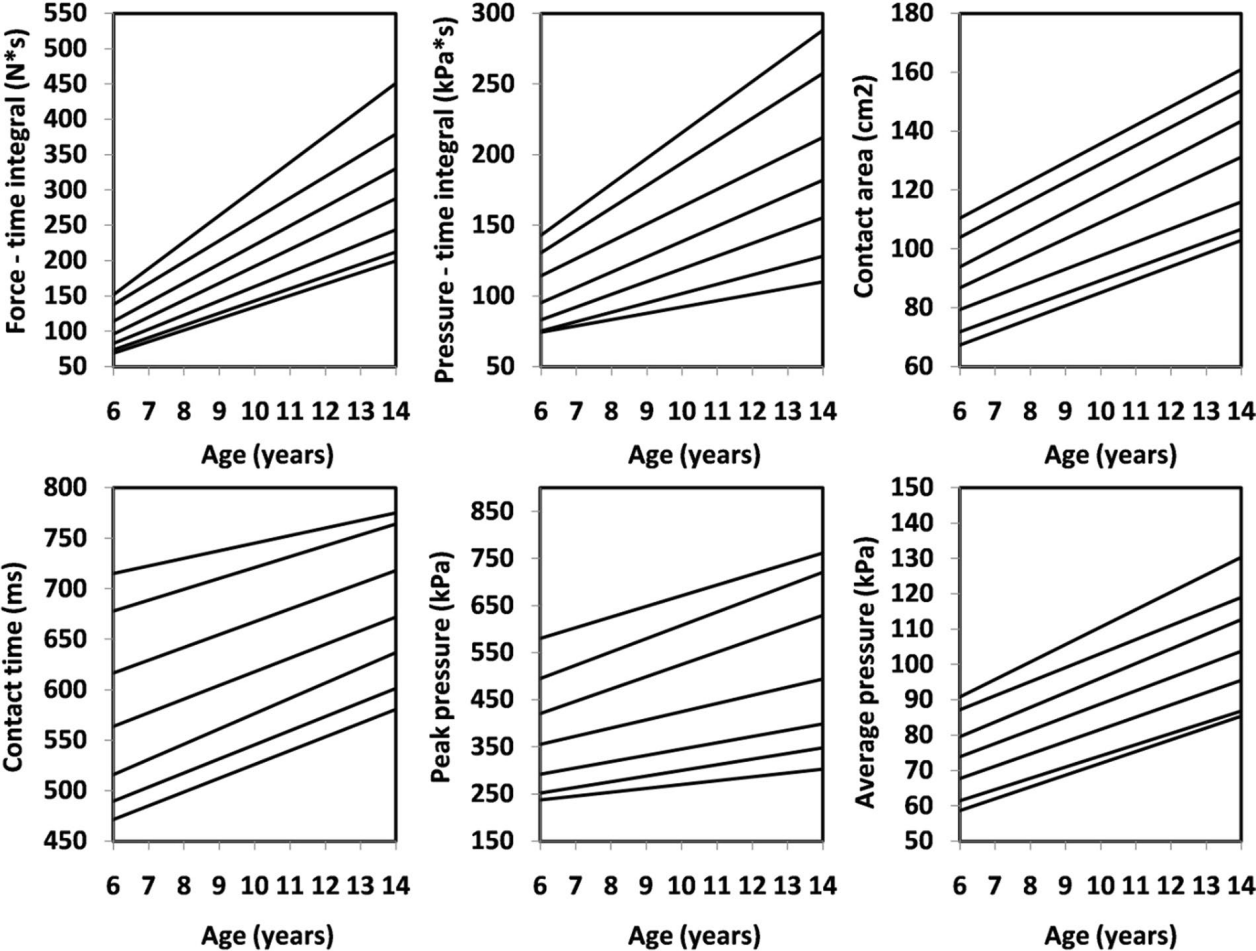
Percentile values for foot function (right) in boys.

**Figure 3:**
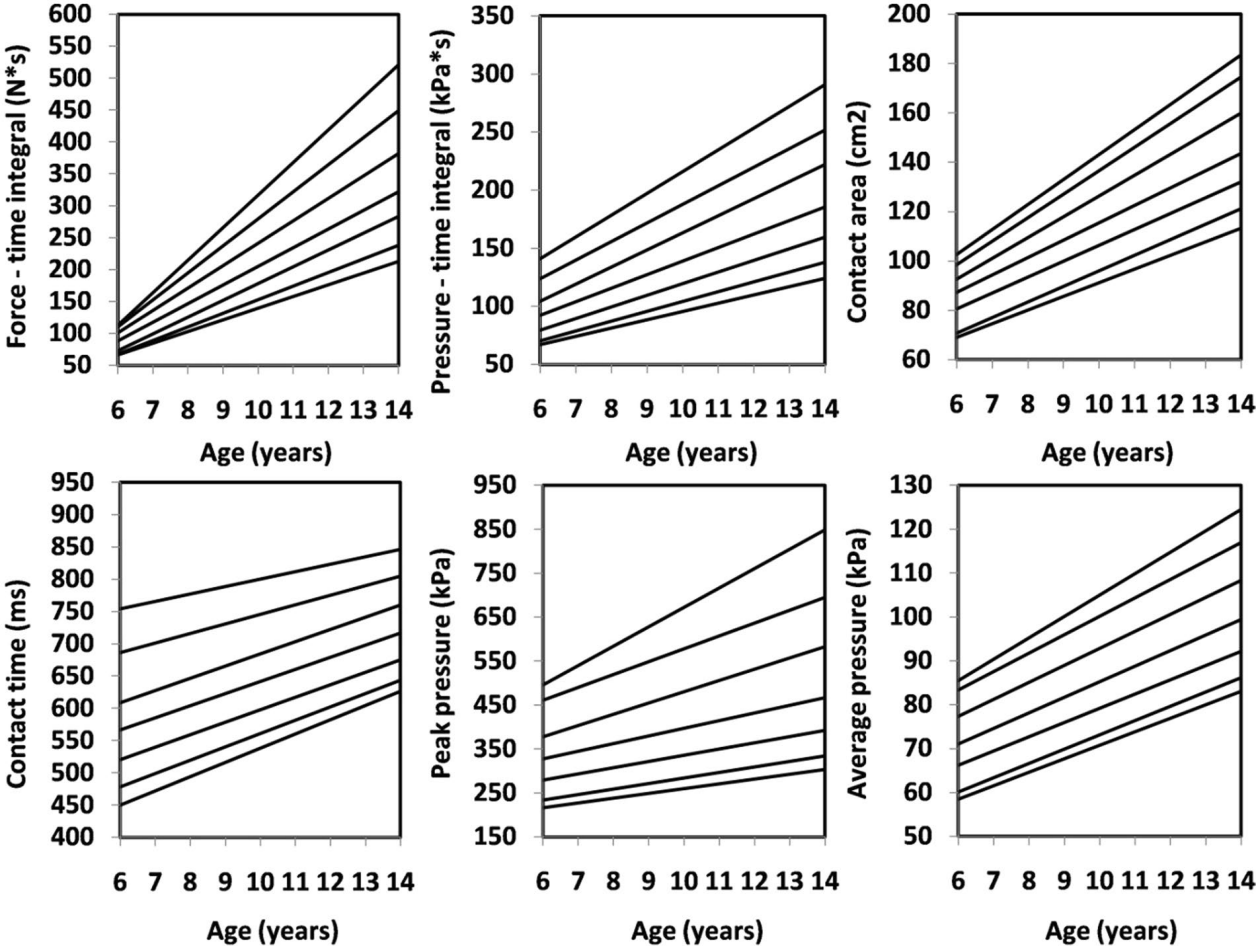
Percentile values for foot function (left) in girls.

**Figure 4:**
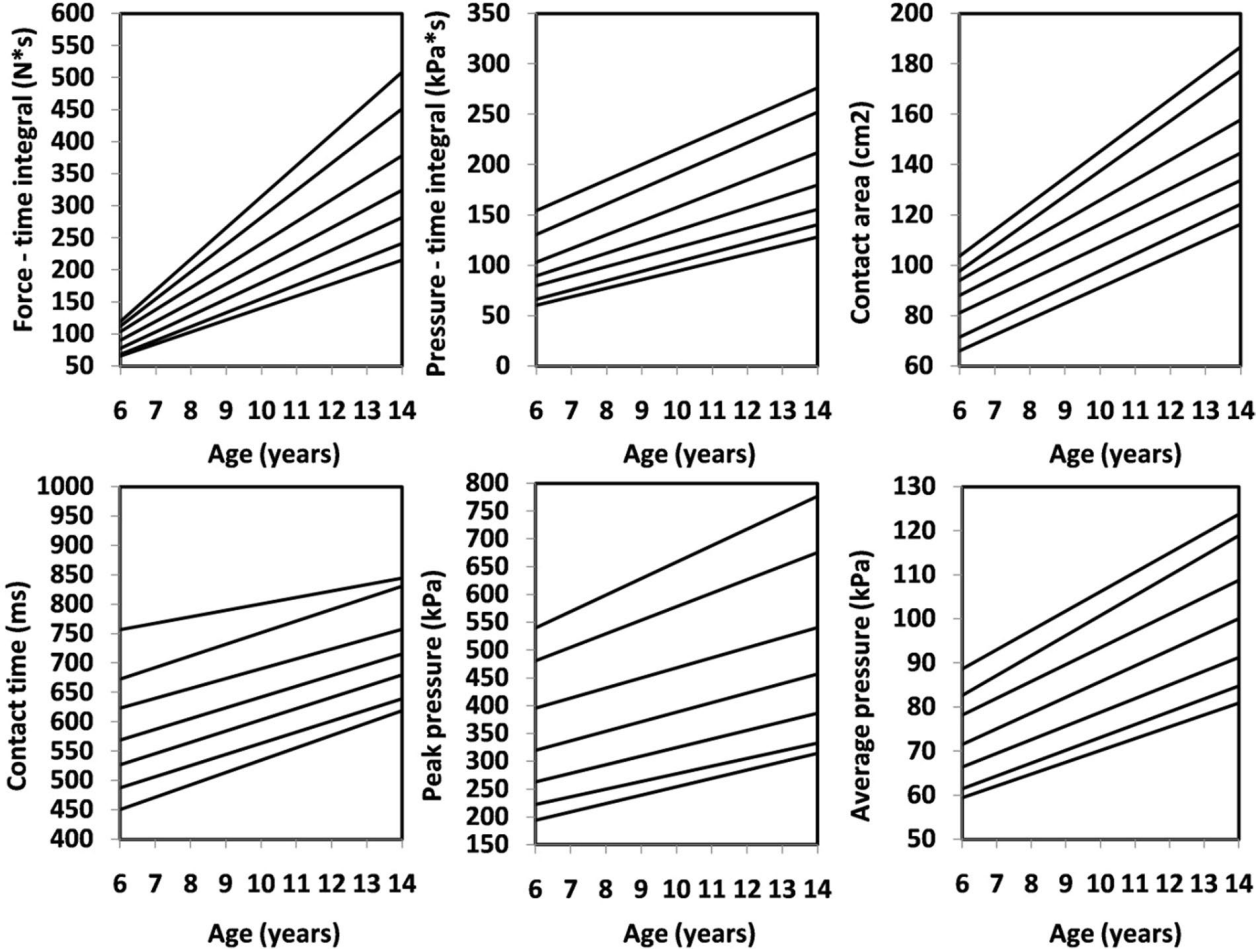
Percentile values for foot function (right) in girls.

For all the data, sex- and age- specific foot characteristics data for force-time integral and pressure-time integral, contact area and contact time and peak pressure and average pressure for left (Fig. 1) and right (Fig. 2) foot in boys and for left (Fig. 3) and right (Fig. 4) foot in girls were created.

## Discussion

The main purpose of the study was to establish sex- and age-specific foot characteristics data in 6–14-year-old children. Of note, this is the first study exploring the aforementioned purpose on a sample of primary school children. The main findings of this study are: (1) boys had higher force-time integral, larger contact area and longer contact time values, compared to girls, (2) girls generated higher peak and average pressures, compared to boys and (3) older children had higher values in all the study variables, compared to younger children.

The biological growth of the structure and shape of a child’s foot is governed by soft tissue, followed by rapid transformation of cartilage develops into bone^19^. The foot is characterized by a highly unique structure which, in addition, can perform diverse movements^20^. However, a deviated foot; i.e. a foot that generates higher plantar pressure may potentially cause discomfort or pain^21^. Previous studies have reported that a few modifiable factors, like physical activity^20,22^ may influence on plantar pressure distribution in children. Specifically, a study by Mickle *et al*^20^. showed that higher levels of plantar pressure under the heel region were associated with lower total level of physical activity and less time spent in moderate-to-vigorous physical activity in boys, while in girls, higher levels of plantar pressure under the toe region spent the significant amount of time in sedentary behaviors. Similar findings were reported in a study conducted among overweight children, where higher levels of plantar pressure beneath the forefoot region were significantly correlated with moderate-, vigorous- and moderate-to-vigorous physical activity^22^. Beside physical activity, studies conducted among adults have shown that some of the factors of plantar pressure during walking include prominent metatarsal heads in the forefoot model, Charcot deformity in the midfoot model and hammer toe deformity in the lesser toes^23^. In children, one previous study has shown that peak plantar pressures and percentage of body weight supported (contact area) are significantly higher in children affected by the disease^24^. However, no study to date has systematically established significant factors associated with several foot functions in children and adolescents and apparently healthy individuals. Indeed, previous studies conducted among older adults have shown, that fallers had a significant higher prevalence of foot pain and generated a significantly higher peak pressure and pressure-time integral under the foot, compared to non-fallers^25^, leading to risks of chronic degenerative diseases, disabled range of motion and premature death.

In general, children’s’ feet are significantly different compared to adults’ feet. Specifically, they suffer from more foot pain, due to pediatric fat pad under the midfoot region, which protects excessive pressure^26,27^. Also, deviated foot functions in children are hypothesized to be associated with foot discomfort in adulthood^26^, leading to the conclusion that gait patterns established in childhood often persist later in life. Indeed, studies have shown that the measurement of plantar pressure during dynamic conditions is considered a reliable method to evaluate foot geometry and function^27^. Since previous evidence has shown that such conditions can be corrected, modifiable risk factors for plantar pressure, such as physical activity, should be organized for a ‘risky’ group of children and for those with deviated foot functions to prevent from future diseases.

Our study has several strengths. First, we collected the data from a large sample of 6–14-year-old children (N = 1 284). Second, we used an objective measure to assess several plantar pressure distribution variables. Third, we presented the results specifically for left and right foot, as preliminary analysis showed significant differences between them.

However, our study has a few limitations. First, foot characteristics in growing children and adolescents should be obtained from longitudinal studies that give the possibility to assess natural changes in individual growth and development^28^. Second, we did not collect the data regarding different foot regions, like heel, midfoot (medial and lateral), forefoot (medial and lateral), toes 2–5 and 1^st^ toe, like done in previous studies in children^8,20,22^. Previous evidence suggests that foot parameters need to be determined under specific regions of interests, in order to detect clinically relevant data to prevent developing foot pathologies in the future^29^. However, if EMED® platform is used to assess foot function beneath different foot regions, future results can be comparable to ours by calculating the overall mean of a given variable. Although we did not collect the data regarding the structure and different foot regions, our additional findings show no foot asymmetries between the feet, participants reported no foot pain in the last 30 days and the prevalence of overweight/obesity in our sample was very small (4%), assuming that children and adolescents are apparently healthy individuals with no foot problems. Also, studies have shown that foot flatness significantly decreases after the age of 6^30,31^. Since we based our findings on youth aged 6–14, it is possible that they have already established foot structure and function naturally.

In conclusion, this is the first study establishing sex- and age-specific foot characteristics data for force- and pressure-time integrals, contact area and contact time and peak and average plantar pressure in 6–14-year-old children. Our results should be of extreme interest for health-related professionals, including orthopedics and podiatrists, who can identify children with deviated foot function and give advice for special comfortable shoes or footpads. Also, kindergarten and primary school teachers (especially in physical education) should monitor and track annual foot changes, in order to detect preschool and primary school children who are at extreme risk and should enter special interventions that correct for potential deviations. The reported norms, for example>90^th^ percentiles for each variable studied, can be used as an ‘alert’ with additional lifestyle factors entered as co-morbidities. Also, the results presented in this study were in percentiles, and may help children and adolescents to memorize the score and track the results over a longer period of time.
